# A combination of suprachoroidal injection of triamcinolone using a custom-made needle and intravitreal Ziv-aflibercept every eight weeks to manage naïve/denovo central DME: a single-center retrospective case series

**DOI:** 10.1186/s40942-024-00550-8

**Published:** 2024-04-02

**Authors:** Ameen Marashi, Marwa Baba, Sedra Abu Ghedda, Mohammad Nour Kitaz, Aya Zazo

**Affiliations:** 1 Marashi Eye Clinic, Aleppo, Syria; 2 Faculty of Medicine, University of Aleppo, Aleppo, Syria; 3 Department of Neurosurgery, Aleppo University Hospital, University of Aleppo, Aleppo, Syria

**Keywords:** Custom-made needle, DME, Triamcinolone, Suprachoroidal, Ziv-aflibercept

## Abstract

**Background:**

Previous studies have shown promising effects of combining intravitreal bevacizumab and suprachoroidal injection of triamcinolone acetonide in treating DME. However, further research is needed.

**Objective:**

To assess the efficacy and safety of combining both intravitreal Ziv-aflibercept and suprachoroidal injection of triamcinolone acetonide using a custom-made needle in naïve and de novo central diabetic macular edema (DME) patients every eight weeks for 24 weeks.

**Methods:**

Central macular thickness was measured via spectral domain-optical coherence tomography, and best-corrected visual acuity was measured via a Snellen chart at baseline and at 4, 8, 12, 16, and 24 weeks postinjection. Additionally, cataract progression, intraocular pressure (IOP), and ocular safety were analyzed.

**Results:**

A total of 10 eyes of 6 patients were treated with suprachoroidal injections of triamcinolone acteonid combined with an intravitreal injection of Ziv-aflibercept. Vision improved from 0.69 log minimum angle of resolution (MAR) at baseline to 0.39 log MAR after treatment. Central macular thickness significantly decreased from 462.3 ± 166 μm at baseline to 362.7 ± 77.6 μm at 24 weeks postinjection.

**Conclusion:**

Suprachoroidal injection of triamcinolone using a custom-made needle with the intravitreal agent Ziv-aflibercept to treat de novo/naïve central DME has favorable outcomes and adequate safety results. Moreover, this study demonstrated the benefit of adapting the previous treatment combination for extending the interval between anti-VEGF treatments from 4 to 8 weeks, which could prevent further expenses, especially in low-income countries.However, large multicenter randomized clinical trials with longer follow-up periods are needed to assess this treatment route, especially in low-income and resourced countries.

## Introduction

Diabetic macular edema (DME) is one of the leading causes of visual impairment globally [1]. The primary treatments for DME are intravitreal anti-VEGF therapy and steroids, which have been successfully demonstrated to be effective. The vitreous serves as an excellent deposition site for medication, prolonging the half-life of drugs [2].

In patients with naïve or de novo DME, the treatment of choice is four or more consecutive injections of intravitreal anti-VEGF every four weeks, which increases the cost burden on patients. Ziv-Aflibercept, initially utilized in oncology cases (FDA approved since 2012), has found a new avenue in addressing ophthalmic concerns, particularly in regions where the ophthalmic form aflibercept (Eylea) remains financially out of reach. This adaptation of Ziv-Aflibercept, known as Zaltrap by Regeneron Pharmaceuticals, has undergone rigorous examination,demonstrating both its non-toxic nature and its effectiveness and safety in treating DME. [3]. Intravitreal steroids can be administered in two forms: as an intravitreal implant (dexamethasone or fluocinolone) or as a solution, such as triamcinolone, which is used for chronic or persistent cases. However, combining both treatments achieved better anatomical and functional results [4]. Intravitreal steroids may offer extended efficacy [5], which could increase the treatment interval in patients managed with intravitreal anti-VEGF and intravitreal dexamethasone implants [6], thus potentially reducing the cost burden on patients.

However, dexamethasone implants cannot be afforded or accessed by patients in many low-income nations, such as Syria, due to their expensive price tags and sanctions.

The suprachoroidal space serves as a promising pool due to its unique location, being anatomically linked to the choroid and sclera, offering slow washout and enhanced medication absorption into the target tissue.

Pharmacokinetic trials in animal models have shown a 12-fold increase in the concentration of medication in the retina and choroid, with only up to 3% of the drug entering the anterior chamber. This decreases the risk of increased intraocular pressure (IOP), cataract formation, and exacerbation or progression of preexisting glaucoma.

Systemic withdrawal of corticosteroids has also been minimized with the suprachoroidal pathway compared to other ocular injections.

Porcine models have established a tenfold decrease in the dosage necessary for achieving a therapeutic outcome, similar to the intravitreal route [7–10].

Human studies assessing the safety and efficacy of suprachoroidal drug injection have been conducted for several disorders, including DME, macular edema related to retinal vein occlusion, and noninfectious uveitis [11].

A phase 3 trial of suprachoroidal delivery of branded preservative-free triamcinolone acetonide (Clearside Biomedical, Alpharetta, GA, USA) involving a modified system (Xipere; Clearside Biomedical) that gained FDA approval for its efficacy and demonstrated an excellent safety index for noninfectious uveitis, with a rate of increased IOP of 11.5% among patients [12].

Thus, compared with the posterior subtenon or intravitreal routes, the suprachoroidal approach has great potential for delivering therapeutic outcomes with improved hypothetical safety.

This study sought to retrospectively analyze the effectiveness and safety of administering 4 mg/0.1 ml of triamcinolone acetonide into the suprachoroidal space using a custom-made needle in combination with 1.25 mg/0.05 ml of intravitreal Ziv-aflibercept for managing patients with naïve and de novo DME every eight weeks, with a follow-up period of 24 weeks. The objective of this study was to evaluate the potential of this treatment regimen to alleviate patient burden, particularly among those with limited resources.

## Patients and methods

### Study population

This single-center retrospective case series incorporated data from patients with naïve or de novo DME treated with suprachoroidal triamcinolone injection and intravitreal Ziv-aflibercept at Arabi Hospital, Aleppo, Syria, between January 2021 and June 2021.

The inclusion criteria for patient records included central DME with best-corrected visual acuity (BCVA) better than 20/320 and central macular thickness (CMT) > 275 μm; no previous ocular history of glaucoma; no usage of IOP-lowering topical medication; and an IOP < 21 mm Hg.

DME patients who had not received treatment or had previously been treated with an injection or laser for at least four months before the administration of triamcinolone suprachoroidal injection or intravitreal Ziv-aflibercept. The study included a total of 10 eyes of 6 patients, 3 eyes had naive central DME and 7 de novo. All patients developed DME as a complication of diabetes mellitus type 2.

Approval for this retrospective interventional case series study was provided by the Ethics Review Board of Arabi Hospital (serial number 10290). Written informed consent was obtained from all patients, and the need for written informed consent was waived by the ethics committee. The reporting of this study conforms to the STROBE guidelines [13]. Patients or the public were not involved in the design, conduct, reporting, or dissemination of our research.

### Treatment

All eligible patients in the study received established treatment every eight weeks for central DME by injecting 0.05 ml/1.25 mg of Ziv-aflibercept (Zaltrap)and 0.1 ml of 4 mg of triamcinolone acetonide (Tricort, Italy).

The treatment combination was repeated every eight weeks if there was a central macular thickness > 275 μm at eight weeks postinjection based on optical coherence tomography or/and if the BCVA was 20/32 or less. However, treatment is withheld if the BCVA improves to 20/25 or better and/or the CMT is > 250 μm.

A custom-made needle manufactured by Ameen Marashi, MD, was used to inject the needle into the suprachoroidal space by the sole physician Ameen Marashi, MD.

The needle was developed from a short, beveled 1-inch, 30G needle with an envelope obtained from a rubber plastic stopper, allowing only 1000 microns out of the 30-gauge needle to penetrate the suprachoroidal space at the pars plana.

**Fig. 1:**
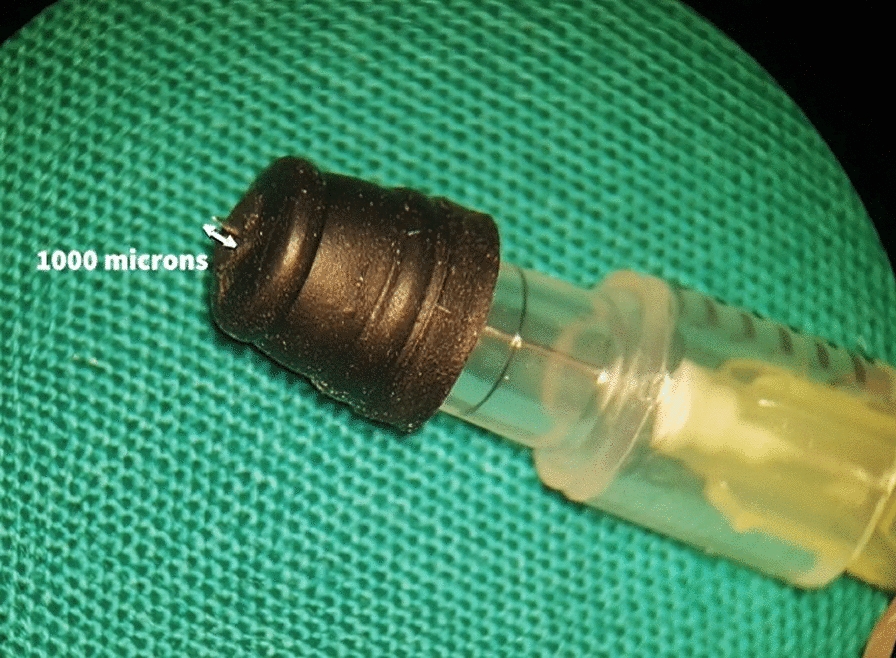
The needle used in the injection. Custom-made and developed by the author

The technique of manufacturing the stopper, mounting it on the needle, and injection techniques are already well described in previous publications and a video published by the author [14–17] Fig 1.

The periocular area was disinfected with 10% povidone-iodine, and a drop of 4% povidone-iodine was introduced into the conjunctival cul-de-sac to disinfect the conjunctiva.

Under topical anesthesia, after using both a drape and an eye speculum, the injection position was marked 4 mm from the limbus in the superior-temporal quadrant.

Then, 0.1 ml of triamcinolone acetonide (4 mg) was injected into the suprachoroidal space using a custom-made needle by keeping the bevel of the 30G needle away from the limbus and inserting it perpendicularly into the sclera until the stopper touched the conjunctiva 17.

No resistance is felt during injection into the suprachoroidal space, and then the needle is obliquely withdrawn to avoid reflux.

Then, 4 mm for phakic patients and 3.5 mm for pseudophakic patients were marked in the inferior-temporal quadrant, 0.05 ml of Ziv-aflibercept (1.25 mg) was injected, and a cotton applicator was used at the injection site to prevent reflux.

Then, the patient's VA and vein pulsation were checked to rule out any retinal artery occlusion; however, patients who had a sustained increase in IOP were managed in situ with anterior chamber paracentesis.

The patient's pupil dilated after injection to assess the peripheral retina to check for any triamcinolone dissemination into the intravitreal cavity, the presence of any suprachoroidal hemorrhage, or choroidal detachment.

### Study outcomes

The primary outcomes were BCVA (by Snellen chart, converted to log MAR for statistical analysis); CMT, measurements taken by spectral-domain optical coherence tomography (SD-OCT) with Nidek RS-330 Retina scan duo (Gamagori, Aichi, Japan) The limit used for macular edema was 275 microns; and IOP, which was recorded at baseline before injection and at monthly postinjection.

### Statistical analyses

The data are presented as the mean ± SD, median (false range), or n (%) prevalence and were statistically analyzed using Microsoft Excel software, 2013 (www.Microsoft.com).

Changes between time points were analyzed by the Wilcoxon signed-rank test, and a P value < 0.05 was considered to indicate statistical significance.

## Results

A total of ten eyes of six patients were eligible for study inclusion (mean age, 61.2 ± 7.48 years). Five eyes received three treatment combinations every eight weeks, and three eyes received only two injections during the 24-week follow-up. However, two eyes received four treatment combinations. All patients were female.

Five eyes were phakic (50%), and five eyes (50%) were right eyes. There were no visually significant lens opacities among the phakic patients.

The mean BCVA (log MAR) improved from 0.69 ± 0.32 at baseline to 0.39 ± 0.2 at 24 weeks (P = 0.005).

The CMT decreased from 462.3 ± 166 μm at baseline to 348 ± 113.9 μm (24.7% reduction; P = 0.023) at 4 weeks post injection, with an additional reduction at week 8 to 351.7 ± 102.5 μm (23.9% reduction from baseline; P = 0.025 versus baseline), and at week 24 to 362.7 ± 77.6 μm (20% reduction from baseline; P = 0.045 versus baseline). Only one eye had increased IOP post-injection and was treated by topical antiglaucoma. Also, one eye only had cataract progression during the follow up period.

**Fig. 2:**
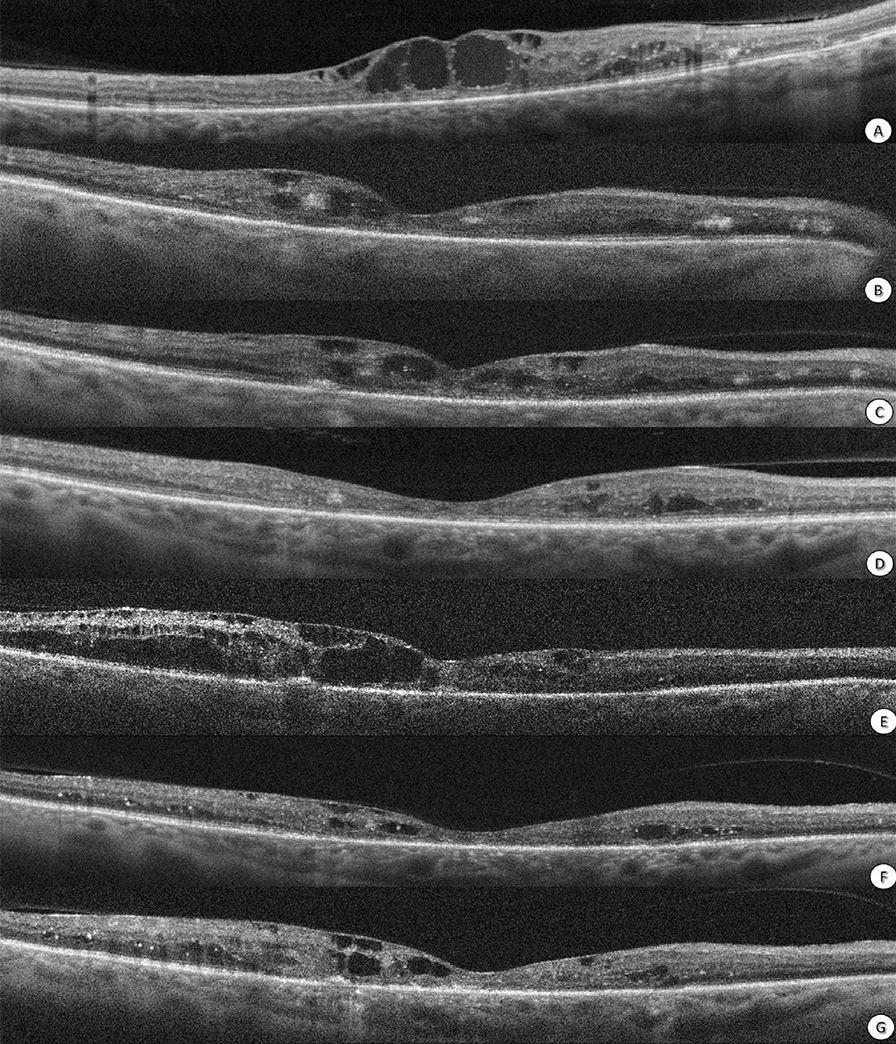
OCT for central diabetic macular edema (DME) treated with a suprachoroidal injection of triamcinolone and intravitreal Ziv-aflibercept. **A** Central DME with increased central macular thickness (CMT) with intraretinal cystic changes and hard exudates. **B** Four weeks after suprachoroidal injection of triamcinolone and intravitreal injection, Ziv-aflibercept reduced CMT and partially resolved intraretinal cysts. **C** Eight weeks after the first injection, the CMT increased, and intraretinal cystic changes reformed. At this point, the injection combination is repeated. **D** Four weeks after the second injection, a reduction in CMT and resolution of central intraretinal cysts were detected. **E** Eight weeks after the second injection, the CMT increased, and central intraretinal cystic changes reformed. At this point, the injection combination is repeated for the third time. **F** Four weeks after the third injection, a reduction in CMT and partial resolution of central intraretinal cysts were detected. **G** Eight weeks after the third injection, the CMT increased, and there was a slight reformation of the central intraretinal cyst

The mean reduction in CMT was 203.8 μm after 6 months in eyes with IOLs compared with eyes without IOLs (− 4.6 μm) (P = 0.004). However, there was no significant difference in BCVA reduction between eyes with IOLs and eyes without IOLs (Fig. 2).

## Discussion

Laser was the primary treatment for DME, employing focal/grid laser techniques, which provided months of DME stabilization with the requirement for robust glycemic control. However, laser treatment can result in laser scars that can cause central scotoma and vision reduction [2].

However, we currently live in the era of intravitreal injections of both AntiVEGF and steroids. Although the former yields better visual and anatomical outcomes than laser treatment [18], it requires four to five injections monthly as a loading dose in de novo or naïve patients, thus requiring compliance from the patient, which may add additional social, professional, and cost burdens.

Ziv-aflibercept (Zaltrap, Sanofi-Aventis US, LLC, Bridgewater, New Jersey, USA) is an active molecule identical to aflibercept (Eylea, Regeneron, Tarrytown, NY) but with a different dose and high osmolarity buffer solution. FDA clearance for recto colon cancer as intravenous treatment. Nevertheless, the safety of the intravitreal agent ziv-aflibercept has been proven in a retrospective multicenter study of 5916 eyes with various retinal diseases, and this agent has side effects similar to those of other intravitreal VEGF blockade agents [19].

Multiple studies have shown the safety and efficacy of intravitreal Ziv-aflibercept for the treatment of DME [3, 20, 21]. Moreover, in a double-blinded, three-arm, randomized clinical trial that compared 1.25 mg, 2.5 mg of Ziv-aflibercept and 1.25 mg of bevacizumab (Avastin; USA; Genentech/Roche) for the treatment of DME in 123 eyes and with a follow-up period of 12 weeks, there was no difference in visual outcomes between the two doses of Ziv-aflibercept. However, they outperformed intravitreal bevacizumab (1.25 mg) in patients who presented with high CMT and low BCVA at baseline. Notably, there was no significant difference between the two doses of Ziv-aflibercept in terms of visual acuity outcomes [22].

Approximately 40% of DME patients are not responsive to anti-VEGF therapy [23]. Therefore, intravitreal steroids can be utilized in chronic or persistent DME [24], as the inflammatory mechanism is the main pathological pathway of DME formation [25].

The MEAD study [26] revealed that an intravitreal implant of 0.7 mg dexamethasone (Ozurdex, Allergan, Irvine, CA) is effective for treating DME for up to six months and has a more favorable safety profile than other steroids. However, there is still a risk of glaucoma and cataract progression. Therefore, it is better to utilize intravitreal steroids in pseudophakic eyes without a history of glaucoma.

In a prospective hospital-based noncomparative study of thirty eyes in thirty patients, a single intravitreal implant of 0.7 mg dexamethasone (Ozurdex, Allergan, Irvine, CA) was used as the initial therapy for the treatment of DME, and patients were followed for one year. One study revealed that an intravitreal dexamethasone implant was safe and effective as an initial treatment option for DME [27].

Several studies have shown the efficacy and safety of utilizing suprachoroidal triamcinolone acetonide injection for DME. For example, in an interventional case series [28], 22 eyes with refractory DME were evaluated. Those eyes were managed with a suprachoroidal injection of triamcinolone acetonide, followed up for three months, and it was concluded that suprachoroidal injection of triamcinolone acetonide for refractory DME is safe and effective.

Another study by the author [29] assessed the safety and efficacy of suprachoroidal injection of triamcinolone acetonide for the treatment of DME every eight vs. 16 weeks. Every eight weeks of suprachoroidal injection yielded better results than every 16 weeks, as 42% of eyes required injection every eight weeks, as CMT was 456 µm pretreatment and decreased to 309 µm at 6 weeks postinjection. Nevertheless, the CMT increased to 384 µm 8 weeks postinjection and decreased again to 330 µm after the second injection. BCVA increased from 20/125 to 20/40 within 16 weeks in these eyes. However, 58% of eyes needed only one injection within 16 weeks; the mean CMT was 421 µm pretreatment and decreased to 339 µm at 6 weeks postinjection, but the central macular thickness increased to 384 µm at 16 weeks. BCVA increased from 20/80 to 20/50 within 16 weeks in these eyes.

Yousef MS et al. [30] also assessed the safety and efficacy of suprachoroidal injection of triamcinolone acetonide for the treatment of DME. It was concluded that suprachoroidal injection of triamcinolone acetonide is safe and effective for treating DME.

Zakaria et al. [31] compared the safety and efficacy of intravitreal and suprachoroidal injections of triamcinolone for DME in a prospective randomized comparative interventional study of 45 eyes of 32 patients divided into three arms: one arm received 4 mg/0.1 ml of intravitreal triamcinolone (group I), one arm received suprachoroidal injection of 4 mg/0.1 ml of triamcinolone (group II), and one arm received suprachoroidal injection of 2 mg/0.5 ml of triamcinolone (group III). It was concluded that suprachoroidal injection of triamcinolone is safe and effective, has effects comparable to those of the intravitreal route. With an advantage of the SC route over the IV which is longer effect on reduction in CMT and improvement of BCVA. They also found that 4 mg does of SCTA is more effective and lasted longer than the 2 mg dose without higher rater of steroids complications.

Lin et al. evaluated [32] in a multicenter analysis of two arms intravitreal aflibercept alone vs. intravitreal aflibercept combined with an intravitreal dexamethasone implant for 102 eyes for 61 patients with DME for six months. They concluded that the use of intravitreal aflibercept with a dexamethasone implant is not inferior to the use of intravitreal aflibercept alone. Nevertheless, the former has a more prolonged treatment interval with superior visual outcomes in pseudophakic patients.

Neto et al. assessed [33] the efficacy of a combination of triamcinolone with bevacizumab vs. monotherapy (bevacizumab or triamcinolone) in a randomized multicenter clinical trial for 111 patients with six months of follow-up and concluded that combined therapy or monotherapy is effective. Nevertheless, arms that received steroids required fewer injections than did those with anti-VEGF alone.

Another study [34] compared posterior subtenon triamcinolone with intravitreal aflibercept for the treatment of resistant DME with intravitreal aflibercept alone and reported that the former group was more effective for treating resistant DME.

In a randomized controlled pilot trial conducted by Fazel et al., the outcomes of treating DME were compared between two groups: one receiving intravitreal bevacizumab alone and another receiving a combination of intravitreal bevacizumab with suprachoroidal injections. The study revealed additive positive effects in the combination group, with improvements observed in both BCVA and retinal anatomical outcomes [35].

The HULK study [36] evaluated the efficacy and safety of suprachoroidal injection of triamcinolone in both naïve patients and those previously treated for central DME in twenty patients. However, all patients received a suprachoroidal injection of triamcinolone at baseline. Naïve patients also received intravitreal aflibercept at baseline. Patients could receive a suprachoroidal injection of triamcinolone as needed, and the mean number of injections was 3.0 at the six-month follow-up. The HULK trial concluded that suprachoroidal injection of triamcinolone is safe, tolerable, and effective with minimal side effects in DME patients, especially in treatment for naïve patients.

In a double-masked, randomized, prospective study, the TYBEE study [37] assessed two groups of patients receiving treatment for DME. The first group received both suprachoroidal triamcinolone and intravitreal aflibercept at baseline and week 12 with monthly sham intravitreal injections.

In contrast, the second group received only monthly aflibercept for 12 weeks via a sham suprachoroidal injection. Then, patients received intravitreal aflibercept as needed at weeks 4, 8, 16, and 20 according to specific criteria. The TYBEE study concluded that there were the same visual benefits of utilizing suprachoroidal injection in conjunction with intravitreal aflibercept and intravitreal aflibercept alone at the 24-week follow-up, with the potential for reduced burden.

Regarding the increase in intraocular pressure (IOP) as a complication of TA injections, it's worth noting that in the HULK study, 10% of patients experienced a rise of 10 mmHg or more, while in the active group of the TYBEE study, this occurred in 15% of patients. Additionally, Zakaria et al. found varying increases: 6.7% in group I, 13.3% in group II, and 6.7% in group III. In our study, compared to group II with the same dosage, only one eye experienced an increased IOP, amounting to 10%.

Another TA complication assessed in our study is cataract. Zakaria et al. reported 3 cases of cataract progression in group I, accounting for 30%, 3 cases in group II, representing 33.34%, and 3 cases in group III, amounting to 25%, with no significant difference observed. However, our study revealed only one eye with cataract progression, constituting 10%. Nonetheless, a longer follow-up period is warranted for comprehensive evaluation.

In this study, the authors assessed the efficacy of utilizing both intravitreal Ziv-aflibercept and suprachoroidal injection of triamcinolone in de novo/naïve central DME patients every eight weeks and followed-up for 24 weeks to reduce the frequency of injection and reduce patient burden. This study revealed that this treatment combination achieved a mean reduction in CMT from 456.45 to 247.63 and a mean improvement in vision from 0.75 to 0.40 within 24 weeks of follow-up with only two eyes; one patient experienced an increase in IOP, which was managed with topical antihypertensive drops, and one eye developed cataracts, which were managed with phacoemulsification. In contrast to the TYBEE study, the combination treatment of suprachoroidal triamcinolone and an intravitreal injection of Ziv-aflibercept (not aflibercept) was given every eight weeks and not every 12 weeks. In addition, in the HULK study, only the treatment-naïve patients received combination treatment at baseline and then suprachoroidal triamcinolone as needed. In contrast, the previously treated DME patients received a suprachoroidal injection of triamcinolone at baseline and then as needed.

## Limitations

Multiple aspects limit the current study outcomes. First, if performed incorrectly, suprachoroidal injections carry a hypothetical risk of choroidal detachment or bleeding. Second, patients who present with a thin sclera may undergo triamcinolone penetration into the vitreous cavity because of the static infiltration depth of the needle. Third, patients required longer follow-up to decisively demonstrate the small incidence of variations in IOP and cataract development. Therefore, the short follow-up period in the current study is a limitation. The current study also included a control or sham group. Additionally, the study was piloted in a single center, which restricts the analysis of the results. Therefore, safety outcomes should be considered cautiously because of the small sample size and short-term follow-up.

A more extensive multicenter prospective randomized study with a larger sample size of patients and longer follow-up duration is required to assess the efficacy and safety of a combination of suprachoroidal injection of triamcinolone and intravitreal Ziv-aflibercept to compare this treatment mode with other treatment modalities, such as posterior subtenon or intravitreal triamcinolone with intravitreal Ziv-aflibercept, or the use of another anti-VEGF agent with a suprachoroidal injection of triamcinolone for de novo or naïve central DME.

## Conclusions

Suprachoroidal injection of triamcinolone using a custom-made needle with intravitreal Ziv-aflibercept to treat central de novo or naïve central DME may reduce central macular thickness and improve visual acuity, with acceptable safety results at the 24-week follow-up, and can be a good management option for countries with limited resources. However, a longer duration of follow-up with a larger multicenter prospective randomized clinical trial is needed to further confirm the safety and efficacy of this treatment combination for de novo/naïve central DME patients. The author also advocates for the adoption of their novel, inexpensive needle designed for suprachoroidal injection of triamcinolone.

## Data Availability

All the data generated or analyzed during this study are included in this published article.

## Abbreviations

DME: Diabetic macular edema
IOP: Intraocular pressure
MAR: Minimum angle of resolution
BCVA: Best-corrected visual acuity
CMT: Central macular thickness
